# Intention to use long acting and permanent contraceptive methods and factors affecting it among married women in Adigrat town, Tigray, Northern Ethiopia

**DOI:** 10.1186/1742-4755-11-24

**Published:** 2014-03-16

**Authors:** Alem Gebremariam, Adamu Addissie

**Affiliations:** 1 Department of Public Health, College of Medicine and Health Sciences, Madawalabu University, Bale-Goba, Ethiopia; 2 School of Public Health, College of Health Sciences, Addis Ababa University, Addis Ababa, Ethiopia

**Keywords:** Long acting and permanent contraceptives, Intention to use, Adigrat town, Ethiopia

## Abstract

**Background:**

Despite the increase in contraceptive use worldwide over the last decade, there is still discrepancy in the need to limit birth and utilization of modern contraceptives specifically long acting and permanent contraceptive methods in sub-Saharan Africa including Ethiopia. Intention to use long acting and permanent methods of contraception is an important indicator of the potential demand for family planning services.

**Objective:**

To assess intention to use long acting and permanent contraceptive methods (LAPMs) and identifying associated factors among currently married women in Adigrat town.

**Methods:**

A community based cross sectional study design complemented with a qualitative method was conducted in three selected Kebeles of Adigrat town. A total of 594 study subjects were interviewed. Systematic random sampling method was used to select study subjects. Quantitative data were analyzed using SPSS version 16. Open code software version 3.6.2.0 was used to facilitate coding of the qualitative data. Factors associated with intention were identified using logistic regression model and content analysis was done on the qualitative data.

**Results:**

Intention to use LAPMs was 48.4%. Intention to use LAPMs was higher among women who knew at least one of LAPMs (AOR = 4.7, 95% CI = 1.58, 14.01) and women who do not want to have birth within the next 2 years (AOR = 1.9, 95% CI = 1.22, 3.13). Intention to use LAMPs was less among women who perceive poor support from their husbands (AOR = 0.2, 95% CI = 0.09, 0.45) and those who perceive LAPMs are harmful for the womb (AOR = 0.24, 95% CI = 0.14, 0.41). Similarly, participants in the focus group discussion have expressed their concern on the return of fertility after using implants or IUCD as well as insertion and removal procedures.

**Conclusions:**

The magnitude of intention to use LAPMs in the study area was low. The main limiting factors were fear of side effect, infertility after LAPMs use, knowledge on LAPMs and perception on partner’s support of LAPMs use. To further promote the use of LAPMs addressing associated misconceptions through effective communication strategies and involving spouses in family planning programs is essential.

## Background

Long acting and permanent contraception methods (LAPMs) are convenient for users and effectively prevent pregnancy and also cost effective for programs overtime. They can result in substantial cost savings for couples, governments, and contribute directly to reaching national and international health goals by providing long lasting contraceptive protection [1]. Contraceptive use has increased worldwide over the last decade [2]. Yet, Africa has high unmet need of family planning (FP). Approximately 25% of women and couples in sub-Saharan Africa (SSA) who want to space or limit their births are not using any type of contraception [3]. Many potential clients in SSA lack information or have misconceptions about LAPMs [4,5].

In a country like Ethiopia with high fertility rate and unmet need of contraceptives, shifting towards LAPMs is an important strategy to ensure continuity of services. But the issue is controversial; the contraceptive method mix is dominated by short term methods like pills and injectables [6-9]. The Ethiopian ministry of health has planned and is working on the provision of all FP methods, especially LAPMs in the lowest service delivery level [10]. Despite the fact that modern contraceptive services are made accessible nearly at all major urban areas in Ethiopia (including the study area, Adigrat town) and in most instances at lower or no cost [11], the utilization and intention to use LAPMs is low [8]. The modern contraceptive prevalence rate among currently married women in Tigray, Northern Ethiopia was 21.2%. It account implants 5.6%, female sterilization 0.3%, and none of them were using IUCD [8].

A given behavior is more likely to occur if the intention to practice is strong, no environmental barriers to perform it, and individual has skills and ability to perform the behavior [12]. Intention to use a method of contraception is an important indicator of the potential demand for FP services [13,14]. Therefore, understanding the characteristics of women with intention to use may provide further insight to demand and future use of LAPMs. In Ethiopia in general and in Tigray regional state in particular, very few studies have ever been conducted on intention to use LAPMs. It is, therefore, essential to examine the magnitude of intention to use and identify the associated factors affecting intention to use LAPMs.

## Methods

A community based cross sectional study complemented by qualitative method was conducted from December 20, 2011 to January15, 2012 in Adigrat town, Tigray, Northern Ethiopia. The total population of the town according to the 2007 census report was 57,572, of which 31,573 (54.8%) were females in all age group [15]. According to the information obtained from the town Health Office, in the town there are six Kebeles with a total of 14,000 households (HH). There were 2 public health centers, 1 public hospital, 7 private clinics, 5 drug shops and 4 rural drug venders in the town during the survey.

A sample of 594 currently married women aged 15–49 years were included in the quantitative method. The sample size was determined using single population proportion formula. It is computed by considering the prevalence of intention to use LAPMs (65.8%) in Butajira town, Gurage zone, Southern Ethiopia, unpublished observations from MPH thesis conducted by Abdi Temesgen; marginal error of 4%, and 95% confidence level. This was found 540 and adding a 10% allowance for a non-response rate, the total sample size was 594.

Out of the total 6 administrative Kebeles in Adigrat town, 3 Kebeles were selected by lottery method. The total sample size was allocated by using proportional allocation to size (PAS) to the total number of HH in the selected Kebeles. The study subjects were selected by systematic random sampling. The sampling interval was 11 obtained by dividing the total HH in each of the selected Kebeles to the allocated sample to each of the selected Kebeles. Every 11th HH with currently married women aged 15–49 years were included. The first HH was chosen at the center of each Kebele by lottery method as a starting point, and then the data collectors were going in the right direction from the first chosen HH until the required sample size for the Kebele was achieved.

Eligibility criterion was currently married women of aged 15–49 years who were resident in the selected Kebeles of the town at least for 6 months. In case of absence of eligible woman in the selected HH, the next HH was taken. If there were more than one eligible woman in the selected HH, one was considered by lottery method for the interview.

For the qualitative method, purposively selected currently married women and currently married men were included in the focus group discussion (FGD). Participants interviewed in the quantitative survey were excluded from the FGD. Age and sex of the participants were considered for grouping of the participants in the FGD. Three sessions of currently married women and 2 currently married men FGD were conducted based on information saturation. Each FGD consisted of seven up to twelve discussants. Additionally, a total of six in-depth interviews were conducted with purposively selected FP service providers to see the perceptions and choice of clients towards the LAPMs. The inclusion criterion was health professionals in the health facilities who offer any of the LAPMs, and available during the data collection period.

The questionnaire was adopted and developed with modification from related studies [8,16]. Both quantitative and qualitative instruments were prepared first in English then translated to the local language (Tigrigna) (see Additional file 1). To check consistency of the translation; retranslation to English was done by other translator. Questionnaire was pre-tested on 5% of the same source population other than the sampled population of the town. Based on the pretest, questions were revised, edited, and those found to be unclear or confusing were modified. Finally, structured closed ended Tigrigna version questionnaire was used for data collection.

Five Tigrigna speaker females who had a minimum 12th grade completed were collected the data. Two diploma nurses supervised the data collectors. They have been trained for two days on the study instrument and data collection procedures. Data were collected through face-to-face interview with the study subjects. Filled questionnaires were checked daily for completeness, legibility and consistency.

Prior to data entry, each questionnaire was given a unique code by the principal investigator. The principal investigator prepared template and entered data using Epi Info version 3.5.1. Ten percent of the entered data were double checked by comparing the entered data with the actual questionnaire. Data were cleaned for missing values by running frequencies and outliers by computing standard scores for the quantitative variables.

Cleaned data were exported from Epi Info version 3.5.1 to Statistical Package for Social Sciences (SPSS) version 16.0 for analysis. Descriptive statistics were computed and presented in the form of texts and tables. A binary outcome variable indicating no intention coded as “0” and intention to use any of the LAPMs coded as “1” was used as the dependent variable. Intention to use LAPMs refers to all currently married women who are not currently using LAPMs but reporting intent to use any of the LAPMs at some time in the future. No intention to use LAPMs refers currently married women who are not currently using LAPMs and stating no intention to use LAPMs or unsure of their intention. Knowledge of LAPMs refers to the participants’ ability to name or recognize at least one of the LAPMs (implants, IUCD, female and male sterilization).

Bi-variate analysis was used to determine the association between different factors and the outcome variable. Multivariable logistic regression was used to identify the relative importance of each predictor to the dependent variable by controlling for the effects of other variables. Those variables which were significant on bi-variate analysis (P-value < 0.05) were entered to multivariable logistic regression analysis [7,9,17,18]. The association between dependent and independent variables was determined using odds ratio (OR) with 95% confidence interval (CI). The level of significance was taken at α =0.05.

For the qualitative method, after selecting the participants, appropriate time and comfortable place of discussion was selected and organized. All the FGDs were moderated by the principal investigator with the assistance of one trained note taker. The in-depth interviews were also undertaken by the principal investigator. The FGDs and in-depth interviews were taped and note was taken.

The collected data were transcribed to Tigrigna on daily basis and translated to English for further processing. The principal investigator and note taker reviewed key terms in Tigrigna and their respective translations to ensure a degree of standardization. Final transcripts were compared against note takers’ notes to ensure quality. Open code software version 3.6.2.0 [19] was used to facilitate coding. The various codes were compared based on differences and similarities and sorted in categories. Finally, based on content analysis, the underlying meaning; that is the latent content of the categories were formulated in to a theme [20]. Finally the findings were triangulated with the quantitative result during write up.

Ethical clearance was obtained from Research and Ethics Committee of the School of Public Health, College of Health Sciences of Addis Ababa University (AAU). Written permission letter was also obtained from Tigray Regional Health Bureau, and then Adigrat Woreda Health Office. Oral informed consent was obtained from the study participants. Confidentiality of the participants was kept throughout the study.

## Results

### Socio-demographic characteristics

A total of 591 currently married women were interviewed in the quantitative part. The response rate was 99.5%. The mean age of the participants was 30 (± 6.9 SD) years. Majority of the participants were Tigrie (98.5%) by ethnicity and Orthodox (90.5%) by religion. More than one third of the participants and their partners attended secondary education level while 92(15.5%) of the participants and 54 (9.1%) of their partners had no formal education. More than half of the participants, 345(58.3%), were housewives by occupation where as 158(26.7%) were employed. The mean family size of the participants was 4.5(±1.8 SD). Out of the total participants, 426(72.0%) and 300(50.7%) had television and radio, respectively. About one quarter of the participants have less than 600 Ethiopian birr (ETB) monthly family income. The median monthly family income was 1000.0ETB with the range of 100 to 7000ETB.

Four FGD sessions were conducted. Most (40.6%) of the participants were in the age group of 25–29 years, and almost all were Christian orthodox. More than two third of the participants were housewives by occupation. Thirteen (40.6%) of the participants had 1–2 number of births. Twenty three (71.8%) of the participants were currently using modern contraceptives, dominated by Depo-Provera 12(37.5%). In-depth interview with six FP service providers in the public and also in private health institutions was conducted. All of the participants interviewed were female diploma nurses. Four of the interviewees were trained on insertion and removal of implants, and IUCD.

### Reproductive history of the participants

The mean age of first marriage and first birth of the participants were 19 (±3.3 SD), and 20.7 (±3.2 SD) years, respectively. Forty six (7.8%) of the participants have never given birth. The median number of births was 2 with 1 and 12 minimum and maximum births, respectively. Seventy nine (14.5%) of the participants have experienced child death. Three hundred fifty eight (60.6%) of the participants do not want more child within the next 2 years; 180(50.3%) was to space and 172(48%) was to limit. The mean ideal desired number of births of the participant was 4.26(±1.5 SD). The decision on the number of children to have was decided jointly by the husband and wife in 552(93.4%) of the participants, while 23(3.9%) said wife and 8(1.4%) husband. Five hundred twenty two (88.3%) of the participants discuss about FP with their husband.

### Comprehensive knowledge on modern contraceptive methods and method mix

Five hundred eighty seven (99.3%) participants mentioned (spontaneously or prompted) at least one type of modern contraceptive. Depo-Provera was the first (99.8%), followed by pills (99.5%) to be recognized as modern contraceptive. Of the total participants, 556(94.7%) knew at least one method of LAPMs. The most known method was implant (99.5%), followed by IUCD (88.7%). The least known methods were tubal legation (52.5%) and vasectomy (23.7%) (Table 1).

**Table 1:** Knowledge of modern contraceptives of married women aged 15–45, Adigrat town, Northern Ethiopia, January 2012

**Variables**	**Frequency (n)**	**Percentage (%)**
*Types of modern contraceptives known (n = 587 each)		
Pills	584	99.5
Depo-Provera	586	99.8
Implants	553	94.2
IUCD	493	84.0
Tubal ligation	295	49.7
Vasectomy	132	22.5
Condom	518	87.5
**Source of first information on FP (n = 587)**		
Neighbours/friends/relatives	71	12.1
Health professionals	359	61.2
Mass media	94	16.0
Husband	10	1.7
School	53	9.0
**Know at least one LAPMs (n = 587)**		
Yes	556	94.7
No	31	5.3
*Type of LAPMs known (n = 556 each)		
Implants	553	99.5
IUCD	493	88.7
Tubal ligation	292	52.5
Vasectomy	132	23.7
**Exposure to LAPMs message (n = 556)**		
Yes	488	87.8
No	68	12.2
**Type of media (n = 488)**		
Television	417	85.4
Radio	37	7.6
Print media	34	7.0
*General uses of LAPMs (n = 556 each)		
Prevent unwanted pregnancy	520	93.5
Prevent maternal and child death	342	61.5
Limiting family size	438	78.8
Child spacing	516	92.8

*Each of the percentages does not add up to 100.0 because respondents could choose several responses which could be spontaneous or prompted.

Among the participants, 473(80.0%) have ever used at least one of the modern methods of contraception. The current modern contraceptive use was 51.3% (95% CI = 47.2, 55.4). The most preferred method was Depo-Provera 207(68.3%) followed by pills 35(11.6%). The prevalence of LAPMs use among the women currently taking modern contraceptives was 59(19.5%), and the highest was implants 31(10.2%).

In the qualitative study, it was found that the majority of participants in the study were aware of long-acting methods of contraception (implants, and IUCD). They also recognized the long term protection of pregnancy by avoiding repeated visit for short term contraceptives. But they had limited knowledge of permanent methods; and relied heavily on negative and second-hand stories from friends. There was a strongly expressed fear of procedures, misunderstanding of procedures, and side effects like headaches, bleeding changes, and weight gain.

One FGD participant said that, *“I did not have any knowhow on male and female sterilization, how is that? Implant is very dangerous, I myself saw it in my friend I feared it very much. I know that it suffers you. Sterilization is also harm full, I want to do that but I fear it because I heard that it can harm you if you do not eat well. I want to stop birth but it is said that it is not good for your health and you should eat. Poor who can’t eat well; how can they use it?”* (30 years old women, illiterate, para 5, Depo-provera user).

### Participants’ perception on modern contraception

Eighty one percent of the participants perceive that their husband supports the use of LAPMs. Almost all of the participants (99%) belief that child spacing protects mother’s and child death. Five hundred fifty two (93.4%) of the women perceive that they have access to choice of all methods and facilities with competent providers in their vicinity. Ten percent of the participants do not agree that providers can be trusted to maintain confidentiality; to advice on method use and side effects. More than one quarter (28.9%) of the women perceives that the use of contraceptive is husband’s decision. More than one forth (26.2%) of the participants belief that contraceptives can harm a woman’s womb, especially LAPMs can be very dangerous (Table 2).

**Table 2:** Perceptions on modern contraceptives among married women aged 15–49, Adigrat town, Northern Ethiopia, January 2012 (n = 591)

**Statements regarding perceptions on modern contraceptives**	**Level of agreement**		
	**Agree, n (%)**	**Neutral, n (%)**	**Disagree, n (%)**
Your husband supports LAPMs use	478(80.9)	44(7.4)	69(11.7)
Child-spacing protects mother’s and child death	585(99.0)	5(0.8)	1(0.2)
I have access to choice of all methods, and facilities with competent providers	552(93.4)	28(4.7)	11(1.9)
Providers can be trusted to maintain confidentiality, to advise on method use and side effects	530(89.7)	40(6.8)	25(3.6)
I can discuss about FP with spouse or convince spouse to use contraceptives	556(94.1)	20(3.4)	15(2.5)
Husband decides if wife wants to use contraceptives	171(28.9)	45(7.6)	375(63.5)
Contraceptives can harm a woman’s womb, LAPMs can be very dangerous	155(26.2)	103(17.4)	333(56.3)

Both women and men FGD discussants said that couples should support each other and contraceptive use should be decided together by the couples. But they still have concern about negative effect on the return of fertility after taking implants or IUCD, insertion and removal procedures and effect on physical activities. They were also associated with the type of food they were taking. They had fear of resistance to remove implants by the FP service providers. Some women had concern on IUCD such as the need for a vaginal examination, discomfort during sex, side effects (infection), effects on long-term fertility, and lack of protection against sexually transmitted infections. The FP providers in the in-depth interview said that, the most frequently mentioned attitudes that women had which contribute to keeping LAPMs use low were fears and rumors about the methods, mistrust towards the providers on removal especially implants, and preference for short acting methods.

One FGD participants said that, *“After using IUCD, you may not give birth even it is said that it can stop birth. IUCD can result in sterility. Implant also has pain and may be difficult to remove it if it is absorbed (covered) by fat. Implant can make the injected hand numb and you can’t rise and carry heavy things by that hand.”* (37 years old woman, illiterate, para 9, Depo-Provera user).

### Intention to use long acting and permanent methods (LAPMs)

The prevalence of intention to use LAPMs was 48.4% (95% CI = 44.1, 52.7) while 78(14.6%) participants were unsure of their intention. Of those who had intention, 152(58.9%) had intention to use one of the LAPMs within the next one year. The most preferred method participants’ intend to use was implants 184(71.3%), followed by IUCD 62(24.0%). The main reasons stated for not intending to use LAPMs were fear of side effect (34.5%) and fear of infertility after use (21.1%). Very few women, (1.5%) reported that LAPMs was against religious or cultural beliefs.

The participants in the FGD and in-depth interview with FP providers pointed out that the public might be interested in LAPMs in the future. All providers believe that demand for LAPMs had increased since they had been started to provide in their particular clinic or health center; especially implants are becoming more popular. But the inclusion of LAPMs especially permanent methods (tubal ligation and vasectomy) in the counseling of methods was still not well done. They indicated the need of education and proper counseling services on all methods especially LAPMs and avoid the women’s fear of removal of implants after insertion.

### Factors associated with intention to use LAPMs

Participants whose partners have completed primary education (AOR = 3.7, 95% CI = 1.47, 9.40), and secondary education (AOR = 2.9, 95% CI = 1.13, 7.44) were significantly associated with intention to use LAPMs (Table 3). Women who were employed (AOR = 0.4, 95% CI = 0.23, 0.81), and merchants (AOR = 0.3, 95% CI = 0.10, 0.79) have lower odds of intention to use LAPMs compared to women who were housewives (Table 3).

**Table 3:** Multivariable logistic regression analyses of selected factors affecting intention to use LAPMs among married women, Adigrat town, January 2012

**Variables**	**Total (n)**	**Intention to use LAPM**		**COR (95% CI)**	**AOR (95% CI)**
		**Yes, n (%)**	**No, n (%)**		
**Age (years)**					
15–19	9	6(66.7)	3(33.3)	1.0(ref.)	
20–24	122	58(47.5)	64(52.5)	0.4(0.10, 1.89)	0.4(0.08, 2.69)
25–29	162	82(50.6)	80(49.4)	0.5(0.12, 2.12)	0.4(0.07, 2.25)
30–34	98	46(46.9)	52(53.1)	0.4(0.10, 1.87)	0.2(0.05, 1.68)
35–39	93	45(48.4)	48(51.6)	0.4(0.11, 1.98)	0.4(0.07, 2.87)
40–44	31	17(54.8)	14(45.2)	0.6(0.12, 2.87)	0.7(0.10, 5.35)
45–49	18	4(22.2)	14(77.8)	0.1(0.02, 0.84)	0.1(0.01, 1.09)
**Participant education**					
No education	86	25(29.1)	61(70.9)	1.0(ref.)	
Primary (1–8th)	174	102(58.6)	72(41.4)	3.4(1.98, 6.01)	1.7(0.84, 3.41)
Secondary (9–12th)	192	102(53.1)	90(46.9)	2.7(1.60, 4.76)	1.6(0.73, 3.48)
Higher education	81	29(35.8)	52(64.2)	1.3(0.71, 2.60)	1.4(0.49, 4.02)
**Partner’s education**					
No education	51	10(19.6)	41(80.4)	1.0(ref.)	
Primary (1-8th)	131	71(54.2)	60(45.8)	4.8(2.24, 10.5)	3.7(1.47, 9.40)**
Secondary (9-12th)	198	112(56.6)	86(43.4)	5.3(2.53, 11.2)	2.9(1.13, 7.44)*
Higher education	153	65(42.5)	88(57.5)	3.0(1.41, 6.48)	1.8(0.65, 5.31)
**Participant occupation**					
Housewife	320	175(54.7)	145(45.3)	1.00(ref.)	
Employed	129	45(34.9)	84(65.1)	0.4(0.29, 0.67)	0.4(0.23, 0.81)**
Daily labourer	56	31(55.4)	25(44.6)	1.0(0.58, 1.81)	0.6(0.33, 1.31)
Merchants	28	7(25.0)	21(75.0)	0.2(0.11, 0.66)	0.3(0.10, 0.79)*
**#Family income**	533	258(48.4)	275(51.6)	1.0(1.00, 1.00)	1.0(1.00, 1.00)
**Wants more child with in 2 years**					
Yes	223	87(39.0)	136(61.0)	1.0(ref.)	1.0(ref.)
No	310	171(55.2)	139(48.8)	1.9(1.35, 2.72)	1.9(1.22, 3.13)**
**#Ideal desired no. of births**	533	258(48.4)	275(51.9)	0.7(0.65, 0.84)	0.7(0.62, 0.88)***
**Discussion on FP**					
Yes	467	239(51.2)	228(48.4)	2.6(1.47, 4.55)	1.2(0.58, 2.83)
No	66	19(28.8)	47(71.2)	1.0(ref.)	1.0(ref.)
**Know LAPMs**					
Yes	498	251(50.4)	247(49.6)	3.5(1.47, 8.23)	4.7(1.58, 14.01)**
No	31	7(22.6)	24(77.4)	1.0(ref.)	1.0(ref.)
**Ever use of MC**					
Yes	420	215(51.2)	205(48.8)	1.7(1.11, 2.61)	1.4(0.78, 2.57)
No	113	43(38.1)	70(60.9)	1.0(ref.)	1.0(ref.)
**Current user of MC**					
Yes	245	134(54.7)	111(45.3)	1.6(1.13, 2.25)	0.86(0.53, 1.39)
No	288	124(43.1)	164(56.9)	1.0(ref.)	1.0(ref.)
**Husband support LAPM use**					
Agree	425	232(54.6)	193(45.4)	1.0(ref.)	1.0(ref.)
Neutral	43	14(32.6)	29(67.4)	0.4(0.20, 0.78)	0.8(0.35, 2.00)
Disagree	65	12(18.5)	53(81.5)	0.18(0.09, 0.36)	0.2(0.09, 0.45)***
**Husband decides to use contraceptives**					
Agree	160	68(42.5)	92(57.5)	1.0(ref.)	1.0(ref.)
Neutral	44	11(25.0)	33(75.0)	0.4(0.21,0.95)	0.6(0.27, 1.68)
Disagree	329	179(54.4)	150(45.6)	1.6(1.10, 2.36)	1.0(0.62, 1.69)
**LAPMs can harm womb**					
Agree	146	39(26.7)	107(73.3)	0.19(0.12,0.29)	0.24(0.14, 0.41)***
Neutral	100	30(30.0)	70(70.0)	0.2(0.13, 0.36)	0.26(0.13, 0.49)***
Disagree	287	189(65.9)	98(34.1)	1.0(ref.)	1.0(ref.)

*Significant at P < 0.05, **significant at P < 0.01, ***significant at P < 0.001.

#family monthly income and number of ideal desired children to have in life were treated as continuous variable. no. = number; ref = reference; MC = modern contraceptives.

There was statistically significant association of intention to use LAPMs among those who knew at least one method of LAPMs (AOR = 4.7, 95% CI = 1.58, 14.01) compared to their counterparts (Table 3).

Women who perceived that their husbands do not support the use of LAPMs had 80% lower intention to use LAPMs (AOR = 0.2, 95% CI = 0.09, 0.45) compared to their counterpart (Table 3). Participants’ who perceive LAPMs could harm a woman’s womb had 76% lower odds of intention to use LAPMs (AOR = 0.24, 95% CI = 0.14, 0.41) compared to those who did not perceive it (Table 3).

Prior experience of contraceptive use, current contraceptive use and perceiving husband decides contraceptive use had not significant association on intention to use LAPMs after controlling for other variables (Table 3).

## Discussion

In this study the magnitude of intention to use LAPMs was 48.4%. This result was higher than the finding in Goba town (27.3%), which is found in South East Ethiopia [6]. This discrepancy could be explained by the difference in the study areas, and access to information and the services. More than sixty percent of the respondents did not want to have child within the next two years but their intention was low. This indicates that the FP providers need to assess the reproductive intention of the women and inform them about all the FP methods available during FP counseling. Fifty nine percent of the participants have intention to use at least one of the LAPMs in the coming one year. The most preferred LAPMs was implants (71.3%). This is relevant for program planning and provides some background for the provision of the different contraceptive method choices.

Almost all of the participants (94.7%) were able to recognize at least one of the LAPMs. This finding was higher than the findings in Mekelle (63.9%) [9] and Goba town (66.9%) [6]. This could be due to the recent continuous advertisement of LAPMs through mass media. The least known method was permanent contraceptives. This was similar with the finding in Mekelle [9]. More than one quarter (26.2%) of the participants perceive that LAPMs could harm the womb. Similarly, 53.6% of married women had negative attitude towards practicing of LAPM in Mekelle [9], and 27.5% women in China believe that sterilization is harmful to health [21]. A qualitative study in Scotland found that IUCD was perceived negatively [4]. The qualitative result in this study also indicated the presence of misinformation, fears and rumors on LAPMs which are keeping many women from intending to use LAPMs.

Partner’s education was one of the most important factors positively related to intention to use LAPMs. Similarly, this was positively associated with contraception utilization in Butajira district [7]. Increasing education might help in discussion on modern contraceptive and would increase knowledge about modern FP methods and hence, increase predisposition to their intention and use of LAPMs.

Women who did not desire additional children within the next two years were more likely to intend to use LAPMs. Similarly, the proportion of women intending to use LAPMs declines with increasing ideal desired number of children. This could be explained by the participants’ fear of fertility return after the use of long acting methods. Desire for more children was significant predictor of intention to use contraceptives [16,22]. Participants’ who knew at least one method of LAPMs had higher odds of intention to use LAPMs compared to their counterparts. Similarly, mothers who had knowledge were more likely to use LAPMs in Mekelle [9].

Women who perceive that contraceptives; especially LAPMs could make a woman sterile or harm her womb had 76% lower odds of intention to use LAPMs. In Pakistan, this perception was significantly associated with lower intention to use female sterilization (OR = 0.70), and IUCD (OR = 0.75) [16]. In this study, participants who perceive that their husbands do not support the use of LAPMs had 80% lower odds of intention to use LAPMs. Similarly, study in Butajira also revealed significant association between men’s support to FP and current use of contraceptive [7].

The study used a mixed design which helps to triangulate the quantitative findings with qualitative findings. However, cause and effect relationship was difficult to establish for the factors dealt in the study since it is cross-sectional study. Although an effort was made to ensure representativeness of eligible currently married women in the town, it does not include non married women or ever-married women. Moreover, the study did not ascertain the providers’ attitudes and behaviors on LAPMs use. Further detailed investigation of the FP service providers’ attitude and behavior on LAPMs, and quality of FP counseling sessions in the town should be conducted.

## Conclusions

Based on the findings of the research it is concluded that the magnitude of intention to use LAPMs in the study area is low (48.4%) and the main reasons to this were fear of side effect and fear of fertility return after use. The principal factors affecting intention to use LAPMs were women’s perception that LAPMs can harm womb, and husband’s support of LAPMs use. Other factors which were significantly associated with intention to use LAPMs were knowledge of any of LAPMs, partner’s educational level, participants working status, women’s desire to have additional children within the next two years or soon, and ideal number of children wanted to have. The study has also clearly evidenced that knowledge of LAPMs, especially permanent method, was low accompanied with misconceptions.

The Federal minister of health, regional health bureau and other responsible organizations should continue the promotion of LAPMs through mass media. The woreda health office should design educational programs that promote and reduce barriers to modern contraceptive use especially LAPMs at community level in the town. The FP services should also be relevant for husbands to participate. Every woman who seeks FP information or services should be counseled on all methods of contraceptives to address misconceptions and fears that exist about LAPMs.

## Competing interests

The authors declare that they have no competing interests.

## Authors’ contributions

AG carried out the conception and designing the study, performed statistical analysis and wrote the manuscript. AA participated in designing the study, analysis, reviewing and editing the final draft of the manuscript. Both authors read and approved the final draft of the manuscript.

## Authors’ information

AG (MPH in Epidemiology), lecturer at Madawalabu University, AA (MD, MPH, MA) working in Addis Ababa University School of Public Health.

## Supplementary Material

Additional file 1 **English version data collection tools.** It includes; Informed consent, Participant’s information sheet, Structure questionnaire, Guideline for FGD, and In-depth Interview Guide. Click here for file
