# Supplementary material for: Intention to use long acting and permanent contraceptive methods and factors affecting it among married women in Adigrat town, Tigray, Northern Ethiopia
Source: Reprod Health. 2014 Mar 16;11:24. doi: 10.1186/1742-4755-11-24 (PMC4007570; doi:10.1186/1742-4755-11-24)
Supplement: Additional file 1 — English version data collection tools. It includes; Informed consent, Participant’s information sheet, Structure questionnaire, Guideline for FGD, and In-depth Interview Guide. [file 1742-4755-11-24-S1.docx]

## English version data collection tools

**Addis Ababa University College of Health Science, School of Public Health**

Survey questionnaire on intention to use long acting and permanent contraceptive methods and factors affecting it among currently married women in the reproductive age (15-49 years) in Adigrat town

Questionnaire ID:------------------------- Kebele No. --------------------------------

**I. Participant’s information sheet**

**Greeting**: Good morning/afternoon

My name is---------------------------------------I am working on behalf of research team (project), which is conducted by Addis Ababa University. I would like to ask few questions which take around 20 munities about knowledge, perception, and intention to use family planning methods especially long acting and permanent contraceptives among married women in the reproductive age. Your genuine responses that you are going to give is very important to identify problems related to long acting and permanent contraceptive methods, and design programs of family planning service in this town and in general to our country. You are selected randomly to be participant of this study if you give me consent after you have understood the following information sheet:

**Title of the study**: Intention to use long acting and permanent contraception methods and factors affecting it among married women in the reproductive age (15-49 years) in Adigrat town.

**Back ground of the study**: Despite of the increase of contraceptive use worldwide over the last decade, there is still discrepancy in the need to limit birth and utilization of modern contraceptives specifically long acting and permanent contraception methods in sub-Saharan Africa including Ethiopia.

**Objective of the study:** To assess the magnitude of intention to use long acting and permanent contraceptive methods and factors affecting it among married women in the reproductive age in Adigrat Town.

**Benefit of the study:** The participants will not gain any direct benefit for being they participated. The result can be used as a baseline for further studies that can be done in this town and identify problems associated with intention to use modern contraceptives specifically long acting and permanent contraceptives in the town so that for planning in the town as well as in our country.

The result will be disseminated to AAU College of Health Science, donor/sponsoring organization (UNFPA), Tigray Regional Health Bureau, and Adigrat wereda health office.

**Risk of the study**: The study has no any risk for the participants and interview will be private to make safe participants from any fear.

**Rights of participants**: Participating and not participation is the full right participants and they can stop participating in the study at any time. They can also skip any question which they want to respond. They can ask any question which is not clear for them.

**Confidentiality:** Any information forwarded will be kept private and her name will not be specified.

**II. Informed consent**

I have read this form or it has been read to me in the language I comprehend and understand all conditions stated above. Are you willing to participate in this study?

1. No (Say Thank you) 2. Yes 🡪 continue your interview

**Name of principal investigator**: Alem Gebremariam;

**Address:** Cell phone: 0910352915; E-mail: [alemg25@gmail.com](mailto:alemg25@gmail.com)

**Name of institution:** Addis Ababa University College of Health Science Research Ethics Committee

**Address**: Addis Ababa, Ethiopia

**Tel. No**: 251-11-553873

Signature of the interviewer certifying that the informed consent has been accepted by the participant __________________________ Date________________

Date of interview (**in Ethiopian calendar**) _______/____/________

Result of interview: 1. Completed 2. Respondent not available

3. Refused. 4. Partially completed

Checked by supervisor, name ____________________Signature ____________Date ________

**III. Structure English version questionnaire**

**Part I: Socio-demographic characteristics of the participants**

| **Q.No.** | **Question** | **Choices** | **Remark** |
| --- | --- | --- | --- |
| 101 | How old are You?(age in years) | Enter _______________ |  |
| 102 | What is your ethnicity? | 1. Tigrie 2. Afar 3. Amhara 4. Oromo   88. Others (**specify**)_______________ |  |
| 103 | What is your religion? | 1. Orthodox 2. Muslim 3. Protestant 4. Catholic   88. Others (**specify**)_________________ |  |
| 104 | What is your educational level? | 1. Can’t read and write 2. Can read and write 3. Grade (1-4th) 4. Grade (5-8^th^ ) 5. Grade (9-12th) 6. Grade 12^+^ |  |
| 105 | What is your partner’s educational level? | 1. Can’t read and write 2. Can read and write 3. Grade (1-4th) 4. Grade (5-8^th^ ) 5. Grade (9-12th) 6. Grade 12^+^ |  |
| 106 | Family size of the respondent? | Enter No____________________ |  |
| 107 | What is your occupation? | 1. House wife 2. Government employee 3. Private employee 4. Daily labourer 5. Farmer 6. Student   88.Other **(specify)_____________** |  |
| 108 | What is your partner’s occupation? | 1. Government employee 2. Private employee 3. Daily labourer 4. Farmer 5. Student   88. Other **(specify)_____________** |  |
| 109 | What is your family monthly income? | ___________Ethio.birr |  |
| 110 | Do you have 1 -Television  2- Radio | 1. Yes 2. No 2. Yes 2. No |  |

**Part II. Reproductive history of the participants**

| **Q. No** | **Question** | **Choices** | **Remark** |
| --- | --- | --- | --- |
| 111 | What was your age at first marriage? | ________years |  |
| 112 | Have you ever give birth? | 1. Yes 2. No | **If “No” skip to Q. 117** |
| 113 | How old were you when you have your first child? | ___________years |  |
| 114 | How many births you give? | Enter No__________ |  |
| 115 | How many of them are alive now? | Enter No__________ |  |
| 116 | How many more children do you want? | Enter No__________ |  |
| 117 | Do you want to have a child within two years (Soon)? | 1. Yes 2. No | **If “Yes” skip to Q.119** |
| 118 | If Q117 is **No**, why? | 1. To space 2. To limit   88. Other **(specify)_____________** |  |
| 119 | How many children do you went to have in your life? | Enter No. ______________ |  |
| 120 | Do you discuss with your partner on family planning methods? | 1. Yes 2. No |  |
| 121 | Who decide/will decide on the number of children you want to have? | 1. Husband 2. Wife 3. Both 4. God 5. Others **(specify)**______________ |  |

**Part III. Knowledge of modern contraceptives of the participants**

| **Q.No.** | **Questions** | **Choices** | **Remark** |
| --- | --- | --- | --- |
| 122 | What type/s of modern contraceptive methods do you know? (**Circle all methods mentioned spontaneously or prompted**) | 1. Pills 2. Injectables 3. Implant 4. IUD 5. Female sterilization 6. Male sterilization 7. Condom   88. Others **(specify)** ________________  99. I don’t know | **If she did not know go to Q133** |
| 123 | From whom do you get information on modern contraceptive methods for the first time? | 1. Neighbours/friends/relatives 2. Health professionals 3. Mass media 4. Husband   88. Others **(specify)** ________________ |  |
| 124 | Do you know about LAPMs (methods used for many years or permanently just after having it once) | 1. Yes 2. No | **If “No” skip to Q 133** |
| 125 | Have you ever exposure to LAPMs message through media within the last 12 months | 1. Yes 2. No | **If “No” Skip to Q128** |
| 126 | If “**yes**” what was the type of media | 1. Television 2. Radio 3. Print media (**specify**)__________________ |  |
| 127 | If “**Yes” to Q124**, which one **(Circle all mentioned)** | 1. Implant 2. IUD 3. Female sterilization 4. Male sterilization   88. Others **(specify)____________**_______ |  |
| 128 | If “Y**es” to Q124**, What general uses of LAPMs do you know? **(Circle all mentioned spontaneously or prompted)** | 1. Helps for prevention of unwanted pregnancy 2. Prevention of possible maternal and child death 3. Limiting family size 4. Child spacing 5. Others **(specify)_________________** |  |
| 129 | What do you know about IUD? **(Circle all mentioned spontaneously or prompted)** | 1. It is very effective 2. It is long term (used for more than 5 years) 3. No effect on breast feeding 4. Not good for female at high risk of sexual transmitted infections. 5. No interference with sexual intercourse 6. Immediately reversible 7. Has minimal side effect 8. Ot 88. Others 88. Others **(specify)** __________________ 9. I don’t know |  |
| 130 | What do you know about implant **(Circle all mentioned spontaneously or prompted)** | 1. It is very effective 2. It is used for long term (up to 5 years) 3. No effect on breast feeding 4. Insertion and removal require minor surgical procedure 5. No interference with daily activity 6. Immediately reversible 7. Has minimal side effect 8. Others **(specify)_____________________**   99. I don’t know |  |
| 131 | What do you know about vasectomy **(Circle all mentioned spontaneously or prompted)** | 1. It is fully effective after 3 months of the operation 2. It is permanent 3. Requires safe and simple procedure 4. Don’t need repeated clinic visit 5. No effect on sexual performance and sensation 6. No known long term side effect 7. Requires counselling and informed consent 8. Others **(specify)___________________** 9. I don’t know |  |
| 132 | What do you know about female sterilization? **(Circle all mentioned spontaneously or prompted)** | 1. It is very effective 2. It is permanent 3. Requires safe and simple procedure 4. Don’t need repeated clinic visit 5. No effect on sexual performance and sensation 6. No known long term side effect 7. Requires counselling and informed consent 8. Others **(specify)_____________________**   99. I don’t know |  |

**Part IV: Mothers beliefs and perceptions on modern contraception**

| **Q.No** | **Statements of perceptions on modern contraceptives** | **Choices** | **Remark** |
| --- | --- | --- | --- |
| 133 | Do you think that your husband support LAPM use? | 1. Agree 2. Neutral 3. Disagree |  |
| 134 | Do you think that child-spacing protects mother’s and child death | 1. Agree 2. Neutral 3. Disagree |  |
| 135 | I have access to choice of all methods, and facilities with competent providers | 1. Agree 2. Neutral 3. Disagree |  |
| 136 | Providers can be trusted to maintain confidentiality, to advise on method use and side-effects | 1. Agree 2. Neutral 3. Disagree |  |
| 137 | I can discuss about FP with spouse or convince spouse to use contraceptives | 1. Agree 2. Neutral 3. Disagree |  |
| 138 | Husband decides if wife can use contraceptives | 1. Agree 2. Neutral 3. Disagree |  |
| 139 | Contraceptives can harm a woman’s womb, LAPMs can be very dangerous | 1. Agree 2. Neutral 3. Disagree |  |

**Part V: Practice of modern contraceptives and intention to use LAPMs**

| **Q.No.** | **Question** | **Choices** | | **Remark** |
| --- | --- | --- | --- | --- |
| 140 | Have you ever used a modern contraceptive method? | 1. Yes 2. No | | **If No skip to Q146** |
| 141 | If “Y**es” Q 140**, what was the method? | 1. Pills 2. Injectables 3. Implant 4. IUD 5. Female sterilization 6. Male sterilization 7. Condom   88. Others **(specify)_____________** | |  |
| 142 | For how long did you use it? (not for female and male sterilization) | Enter ____________(months or years) | |  |
| 143 | Have you ever shifted from one contraceptive method to another? | 1. Yes 2. No | | **If “No” skip to Q.147** |
| 144 | If **Yes Q143,** from which contraceptive to which contraceptive | From__________________  to _____________________ | |  |
| 145 | If **“Yes” for Q143**, Why did you shift from one method to another? | 1. For inconveniency of previous method 2. For the convenience of the new method 3. Due to lack of access to the previous method 4. Due to side effect 5. Need for long acting contraceptive method 6. Provider advised me 7. Partner influenced me 8. Others **(specify)** ____________ | |  |
| 146 | If **No for Q140,** why? | 1. Lack of knowledge 2. Lack of access 3. To get pregnant 4. Fear of infertility 5. Partner disapproves 6. I am infecund 7. Fear of side effect 8. It is sinful to use 9. Cultural taboo 10. Others **(specify)_________________** | |  |
| 147 | Are you using modern contraceptive method now? | 1. Yes 2. No | | **If Yes skip to Q149** |
| 148 | If **Q.147 No**, why? | 1. I am pregnant 2. I want to be pregnant 3. I am on exclusive breast feeding 4. I fear side effect 5. I am infecund   88. Others **(specify)** __________________ | |  |
| 149 | **If “Yes” for Q. 147**, Which method are you using now? | 1. Implant 2. IUD 3. Female sterilization 4. Male sterilization 5. Pills 6. Injectables 7. Condom   88. Others **(specify)** _______________ | |  |
| 150 | **If yes to Q.147,** from where do you get the method you are using? | 1. Health center 2. Government hospital 3. Private clinic 4. Pharmacy (Drug vendor) 5. Health extension workers 6. Shop 7. Friends/ relatives   88. Others **(specify)** ______________ | |  |
| 151 | **If Q.149 (1, 2, 3, 5, or 6)** Does your partner approve you taking of the method? | 1. Yes 2. No | |  |
| **Go to the following if the mother is not taking any one of LAPMs and not infecund** | | | | |
| 152 | Do you/your partner want to use any LAPMs to delay or to avoid pregnancy at any time in the future? | | 1. Yes 2. No 3. I am not sure | **If Yes go to Q.154** |
| 153 | If you are not going to use LAPMs, would you tell me the main reasons? | | 1. fear of side effect 2. Lack of awareness of the LAPM 3. Not my preferred method 4. Little risk of pregnancy 5. To have more children 6. Husband disapproval 7. Religion prohibition 8. Fear of infertility 9. Other **(specify)_________________** |  |
| 154 | If “**Yes” to Q.152**, do you intend to use LAPMs in the next 12 months? | | 1. Yes 2. No 3. I am not sure. |  |
| 155 | If “**Yes” to Q.152**, which one do you want? | | 1. Implant 2. IUD 3. Female sterilization 4. Male sterilization 5. Others (**specify**)____________ |  |

**THANK YOU**

I have finished my interview

If you have any question/concern on LAPMs __________________________________________________________

**Guide line for focus group discussion**

Hello, participant’s good morning /afternoon.

My name is _______________ and my colleague here with me is called ________________. We are a team from Addis Ababa University. This discussion is going to be conducted for assessing the status of FP utilization, specifically long acting and permanent contraceptives. We hope that the discussion we would have with you is very much useful to improve the quality and accessibility of contraceptives especially long acting (implant, and IUD) and permanent (voluntary male and female sterilization) in this area and the whole our country. For this discussion I will raise some point for discussion concerning long acting and permanent contraception’s knowledge, attitude, use and future intention to use. Before that I would like to thank for all of you voluntary participants.

**Instruction**

1. Your presence is very important.
2. We are interested in all of your ideas and suggestions.
3. There are no wrong or right answers.
4. All ideas; both positive and negative to the point of discussion are welcomed.
5. Please feel free to disagree with one another. We would like to have many points of view.

We would like to ask your permission to audiotape your comments and opinions so that we could not miss any of your ideas while trying to take notes. And I assure you that all your ideas are confidential, used for research purpose only. I want our session to be a group discussion, so you need not wait for me to call on you. Please speak one at a time, so that the tape-recorder can pick up every of your ideas and suggestions. We have a lot of points to cover, so I may change the subject or move ahead. Please stop me incase if you want to add something more.

It is very important not to have side conversations because it interferes with individual’s full participation in the group discussion and also posse’s challenges for recording the discussion.

Each participant is asked to introduce herself and tell us something about you.

**Discussion topic for the FGD**

**Date of Focus Group discussion:____________________________**

**Location of Focus Group discussion:_________________________**

**Name of Note Taker:_____________________________________**

1. Warm up question
   1. Current issue on family size
   2. Advantage and disadvantage of many children birth.
2. Discussion about modern contraception
   1. Knowledge of contraception
   2. Knowledge of IUD, Implant, male and female sterilization
3. Preference of modern contraception methods
   1. Short term
   2. Long acting and permanent
   3. Providers counseling, choice and skill
   4. What is your attitude and communities perception concerning the use of LAPMs
   5. What are the advantages /disadvantage of LAPMs over the other?
   6. Do the community /you want to use LAPMs in the future? Which method do you prefer? Why?
4. When should people start to use LAPMs?
   1. Who should use LAPMs?/who should decide the use
   2. Age, Religion
   3. Educational status
   4. Economic status
   5. Marital status
5. Is there any additional idea that you want to add on our discussion on LAPMs and related issues?

**THANK YOU!**

**In-depth Interview Guide**

I want to thank you for taking the time to meet with me today.

My name is **Mr. Alem Gebremariam**. I come from Addis Ababa University School of Public Health and I would like to talk to you about your experiences in the family planning clinic on clients’ utilization and choice of modern contraceptives specifically, on the long acting and permanent methods of contraceptives. I am assessing the women’s intention to use LAPMs and factors affecting it that can be used in future interventions. The interview will take less than an hour. I would like to ask your permission for taping the session because I don’t want to miss any of your ideas and suggestions. Although I am going to take some notes during the session, I can’t possibly write fast enough to get it all down. Because we’re on tape, please be sure to speak up so that we don’t miss your ideas.

All responses will be kept confidential. This means that your interview responses will only be shared with research team members and we will ensure that any information we include in our report does not identify you as the respondent. Remember, you don’t have to talk about anything you don’t want to and you may end the interview at any time.

Are there any questions about what I have just explained?

Are you willing to participate in this interview?

__________________ _____/_____/________

Interviewee signature Date

**In-depth interview topics for the family planning providers**

**Date of In-depth interview**:____/______/________________

**Sex of the interviewee**:_______________________________

**Type of Health facility**:___________________________

**Qualification of interviewee**:_________________________

**Name of Note Taker:**_____________________________

1. Adequately trained, supervised and equipped to counsel and provide long acting and permanent contraceptive methods.
   1. Side effects, counseling
   2. Insertion and removal
2. Preference of modern contraceptives in your clinic
   1. Why
      1. Empowerment (woman decision)
      2. Fear of side effect
      3. Knowledge and perception
      4. Availability
3. Who are the users of LAPMs in your clinic?
   1. Age
   2. Religion
   3. Educational status
   4. Economic status
   5. Marital status
4. What can you say about the future intention of clients to use LAPMs?
5. Do you think that there is a need to improve the service
   1. What would improve the provision of family planning services in particular LAPMs?
6. What additional things can you say about LAPMs?

**Thank You**
